# Antibody Titers and Protection against Omicron (BA.1 and BA.2) SARS-CoV-2 Infection

**DOI:** 10.3390/vaccines10091548

**Published:** 2022-09-17

**Authors:** Chloé Dimeglio, Marion Migueres, Naémie Bouzid, Sabine Chapuy-Regaud, Caroline Gernigon, Isabelle Da-Silva, Marion Porcheron, Guillaume Martin-Blondel, Fabrice Herin, Jacques Izopet

**Affiliations:** 1 CHU Toulouse, Hôpital Purpan, Virology Laboratory, 31300 Toulouse, France; 2 Toulouse Institute for Infectious and Inflammatory Diseases (INFINITy), 31300 Toulouse, France; 3 Occupational Diseases Department, Toulouse University Hospital, 31000 Toulouse, France; 4 UMR1295, Unité Mixte INSERM—Université Toulouse III Paul Sabatier, Centre for Epidemiology and Research in Population Health Unit (CERPOP), 31000 Toulouse, France; 5 Infectious and Tropical Diseases Department, Toulouse University Hospital, 31300 Toulouse, France

**Keywords:** SARS-CoV-2, Omicron, antibodies, protection, public health

## Abstract

The emergence of the SARS-CoV-2 variants of concern has greatly influenced the immune correlates of protection, and there are little data about the antibody threshold concentrations to protect against infection with SARS-CoV-2 Omicron BA.1 or BA.2. We analyzed the antibody responses of 259 vaccinated healthcare workers, some of whom had been previously infected by SARS-CoV-2. The median follow-up was 179 days (IQR: 171–182) after blood collection. We detected 88 SARS-CoV-2 Omicron infections during the follow-up period, 55 (62.5%) with SARS-CoV-2 BA.1, and 33 (37.5%) with SARS-CoV-2 BA.2. A neutralizing antibody titer below 8 provided no protection against a BA.1 infection, a titer of 16 or 32 gave 73.2% protection, and a titer of 64 or 128 provided 78.4% protection. Conversely, the BA.2 infection rate did not vary as a function of anti-BA.2 neutralizing antibody titers. Binding antibody concentrations below 6000 BAU/mL provided no protection against Omicron BA.1 infection, 6000–20,000 BAU/mL provided 55.6% protection, and 20,000 or more provided 87.7% protection. There was no difference in BA.2 infection depending on the binding antibody concentration. Further studies are needed to investigate the relationship between antibody concentrations and infection with the Omicron BA.4/5 variants that are becoming predominant worldwide.

## 1. Introduction

Since December 2020, the coronavirus disease 2019 (COVID-19) pandemic has been dominated by the emergence of severe acute respiratory syndrome coronavirus 2 (SARS-CoV-2) variants of concern (VOCs). The latest to date is the Omicron (B.1.1.529) variant, further divided into distinct sublineages: BA.1, BA.2, BA.3, BA.4, and BA.5. These new variants harboring numerous spike protein mutations, particularly in the receptor binding domain (RBD), have spread rapidly worldwide because they were more transmissible or more prone to immune escape. 

The SARS-CoV-2 pandemic has resulted in substantial morbidity, mortality, and social disruption. However, vaccination and a previous infection provide a degree of protection against symptomatic, severe COVID-19 [1]. The vaccines presently available to protect against such infections all use the ancestral (Wuhan-like) virus or its spike protein as immunogen. 

Serological diagnosis is becoming increasingly important in attempts to understand the extent to which COVID-19 is spreading in the community and to identify individuals who are immunized and potentially “protected” against re-infection. The most widely accepted marker of protection against a SARS-CoV-2 infection is the concentration of neutralizing antibody [2,3] that prevent viral infection mostly by blocking the early step of infection, viral entry, especially in interfering with virions binding to their receptor. There is also a correlation between the concentrations of neutralizing antibody and those of binding antibodies measured by immunoassays that use ancestral strain antigens [4,5,6]. The concentration of anti-spike IgG produced by vaccination or natural infection is associated with protection against infections of SARS-CoV-2 strains that were present before the Omicron variant emerged in November 2021 [7,8,9]. Goldblatt et al. used a random effects meta-analytic approach to calculate protective thresholds in WHO units for ancestral strain SARS-CoV-2 and Alpha (B.1.1.7) of 154 (95% CI 42, 559) and 171 (95% CI 57, 519) anti-S binding antibody units (BAU/mL), respectively [10]. It was consistent with the threshold of 141 BAU/mL conferring more than 89% protection, according to another study [7]. A slightly higher protective threshold of 264 BAU/mL was also proposed [8]. Gilbert et al. reported geometric mean neutralization titers ∼247 IU/mL [9], compared to 1057 IU/mL in Khoury et al. [2], suggesting that different protection thresholds could be obtained depending on the neutralization assay used. 

The emergence of these SARS-CoV-2 variants of concern (VOC) with increased transmissibility and the capacity to escape natural and vaccine immunity [11,12,13,14] has greatly influenced the immune correlates of protection. In late 2020, the Delta variant (B.1.617) was detected in India and spread rapidly worldwide, displacing other variants. Notable mutations in the B.1.617.2 variant included L452R, T478K, and E484Q in the S RBD. The combination of mutations in the Delta variant seems to impart the virus a selective advantage compared to the original virus and other variants, as evidenced by high transmissibility and infectivity, and immune evasion [11]. In late 2021, the B.1.1.529 variant emerged in Southern Africa and contains several mutations present in other variants, such as N501Y (Alpha), E484A~E484K (Beta and Gamma), and T478K (Delta), although in total it has more than 50 mutations with more than 30 identified in the S gene alone [15]. These mutations are associated with enhanced infectivity and transmissibility, and Omicron has also been demonstrated to escape neutralization by monoclonal antibodies, convalescent serum, and post-vaccine antibody [12]. Overall, with the exception of the Alpha VOC, the emerging VOCs have been associated with reductions in neutralizing activity of antibodies derived from previously infected or individuals who have undergone primary vaccination [16,17,18,19]. Evidence based on in vitro neutralization assays suggests that, for immune responses to Omicron in individuals who have already been exposed to ancestral SARS-CoV-2 antigens (whether through infection or vaccination), an Omicron correlate of protection may be higher than for ancestral SARS-CoV-2 or other VOCs, due to the reduced effectiveness of antibodies directed against the spike protein. To that point, Pfizer-BioNTech has reported a 25-fold reduction in neutralization titres against Omicron compared to ancestral SARS-CoV-2 in individuals vaccinated with two doses of BNT162b2 [20]. Studies from South Africa and Germany report a reduction in neutralization up to 41-fold [17], despite two or three doses of BNT162b2 or mRNA-1273 and previous infection. We therefore have determined the antibody threshold concentrations (binding and neutralizing antibodies) needed to protect against infection with SARS-CoV-2 Omicron BA.1 or BA.2.

## 2. Material and Methods

### 2.1. Patients

We analyzed the antibody responses of 259 vaccinated healthcare workers (HCWs), some of whom had been previously infected by SARS-CoV-2. Blood samples were taken 3–6 weeks (median: 4.2 weeks) after their last dose of vaccine (12 September 2021–13 October 2021) and before the Omicron variant arrived in France (December 2021–January 2022). The median follow-up was 179 days (IQR: 171–182) after blood collection. This study was approved by the French Research Ethics Committee Est-III (COVID BioToul, ID-RCB 2020-A01292-37, ClinicalTrials.gov Identifier: NCT04385108).

### 2.2. SARS-CoV-2 Detection, Variant Screening, and Genome Sequencing

Nucleic acids were extracted from nasopharyngeal swab samples on an MGI SP-960 instrument using the MGIEasy Nucleic Acid Extraction kit and amplified with the Thermofisher TaqPath RT-PCR assay (ThermoFisher, Waltham, MA, USA) on QuantStudio 5 Real-Time PCR systems. Positive results were then classified, according to the TaqPath S gene profile, as: S gene target failure (SGTF), S gene target late detection (SGTL), or non-SGTF/SGTL [21]. The SGTL profile was defined as a difference of at least 4 Ct between the N and S genes. The Omicron BA.1 variant was identified by its SGTF/SGTL TaqPath profiles (69–70 S gene deletion). Positive specimens with non-SGTF/SGTL profiles (only samples with N Ct ≤ 30) were then tested using the IDTM SARS-CoV-2/VOC Revolution Pentaplex assay (ID solutions, Montpellier, France). This multiplex RT-PCR assay targets the K417N, L452R, and E484K mutations. The Omicron BA.2 variant was identified by the presence of the K417N mutation. Our VOC screening strategy was validated by sequencing a large number of positive nasopharyngeal samples using the Pacific Biosciences (Pacbio) SMRT System [22]. 

### 2.3. Neutralizing and Binding Antibodies against SARS-CoV-2 Spike Protein

Neutralizing antibody (NAb) titers were measured by end-point dilution using Vero cells (ATCC, CCL-81™) [14] and clinical strains of SARS-CoV-2 Omicron BA.1 (EPI_ISL_10316329) and BA.2 (EPI_ISL_13540703). Anti-spike Immunoglobulin G (anti-S) concentrations were measured with an electrochemiluminescent assay, which is a binding antibody assay based on the RBD of the Spike protein (IgG II Quant, Alinity, Abbott, Sligo, Ireland) [4]. Raw data in AU/mL were converted in BAU/mL by using the conversion factor (0.142) recommended by the manufacturer.

### 2.4. Antibodies against SARS-CoV-2 Nucleocapsid Protein

Anti-nucleocapside (anti-N) antibodies were detected with an electrochemiluminescent assay (SARS-CoV-2 IgG, Alinity, Abbott, Sligo, Ireland). They can differentiate between natural and vaccine-derived sero reactivity.

### 2.5. Statistical Analysis

Correlations were identified using a Spearman test and groups were compared with the Chi^2^ or Fisher’s exact tests. Statistical significance was set at: *p*-value < 0.05.

The anonymized data were analyzed using Stata version 14 (StataCorp LP, College Station, TX, USA) and MATLAB 2018b.

## 3. Results

### 3.1. Population Characteristics and Vaccination Status

The 259 HCWs included 193 (74.5%) women (median age: 41.5, range: 21–61) and 66 (25.5%) men (median age: 41, range 22–61, *p* > 0.05 Wilcoxon rank test). 168 (64.9%) had been infected with SARS-CoV-2 before their vaccination (identified by positive PCR or the presence of anti-N antibodies at baseline). Most of them (152/168, 90.5%) had been given a BNT162b/BNT162b, 2-dose, vaccination schedule, while 16 (9.5%) others had a ChAdOx1-S/BNT162b schedule (Table 1). The majority (148/168, 88.1%) had only two doses of vaccine; 20/168 (11.9%) had a BNT162b booster (third dose) between 15 July and 30 August 2021. The 91 vaccinated HCWs with no previous infection included 8 (8.8%) given a ChAdOx1-S/ ChAdOx1-S schedule, 46 (50.5%) given ChAdOx1-S/BNT162b, and 37 (40.7%) given BNT162b/BNT162b (Table 1). Most of the 91 initially uninfected HCW (75, 82.4%) were given a BNT162b booster following 2-dose vaccination; the other 16 (11.6%) had only a primary vaccination (2 doses).

### 3.2. Occurrence of SARS-CoV-2 Infections

We detected 88 SARS-CoV-2 Omicron infections during the follow-up period, 55 (62.5%) with SARS-CoV-2 BA.1, and 33 (37.5%) with SARS-CoV-2 BA.2. No HCW required hospitalization for their SARS-CoV-2 Omicron infection. 

**Figure 1:**
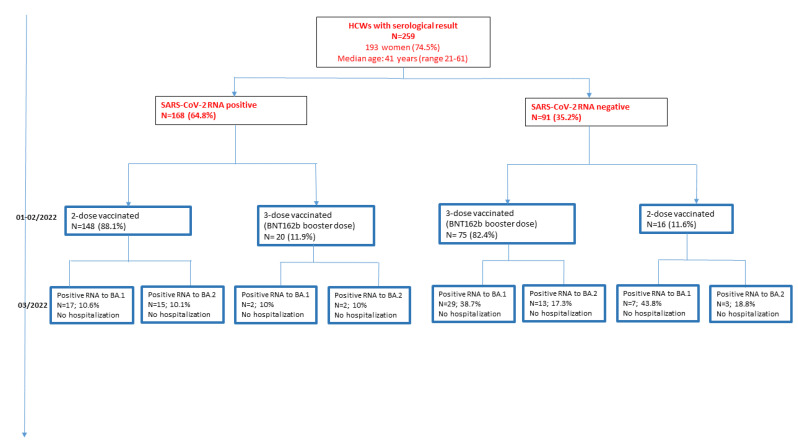
Study flowchart.

The 36 (65.5%) BA.1 infections that occurred in vaccinated HCWs without prior infection included 29/75 (38.7%) detected within 44 days (median) after booster administration (range: 26–118 days), and 7/16 (43.8%) after complete vaccination without booster (Figure 1). The remaining 19 (34.5%) BA.1 infections occurred in HCWs infected with a strain of SARS-CoV-2 before their vaccination: 2/20 (10%) within 50 days (median, range 30–95) after booster administration, and 17/148 (10.6%) after 2-dose vaccination, no booster (Figure 1).

Similarly, 16 (48.5%) uninfected, vaccinated HCWs had BA.2 infections: 13/75 (17.3%) within 44 days (median, range: 17–74 days) after booster administration, and 3/16 (18.8%) after complete vaccination without booster (Figure 1). The remaining 17 (51.5%) BA.2 infections occurred in HCWs infected with a strain of SARS-CoV-2 before their vaccination: 2/20 (10%) within 49 days (median, range 26–169 days) after a booster, and 15/148 (10.1%) after 2-dose vaccination (Figure 1).

### 3.3. Neutralizing Antibodies and Protection against SARS-CoV-2 BA.1 and BA.2

**Figure 2:**
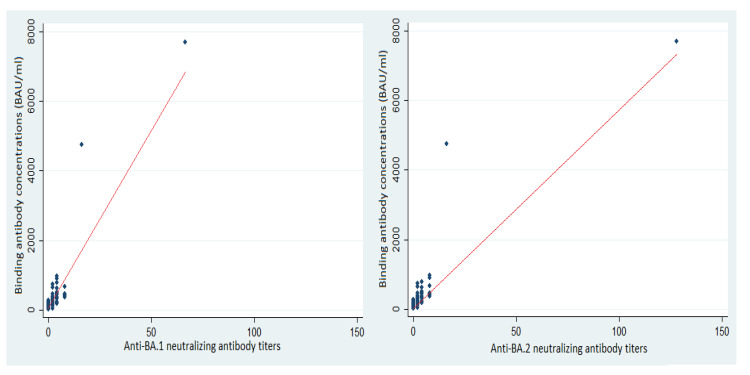
Binding antibody concentrations according to BA.1 and BA.2 neutralizing antibody titers among uninfected/vaccinated HCWs and trend curve (red line).

**Figure 3:**
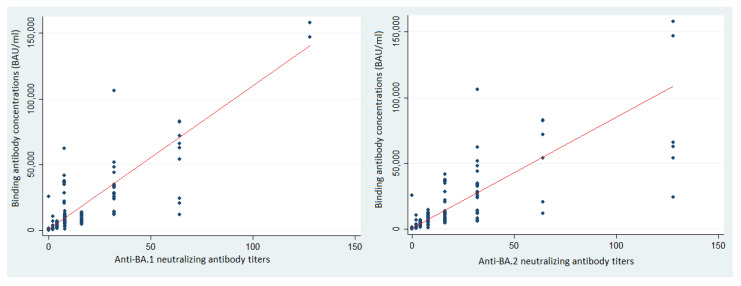
Binding antibody concentrations according to BA.1 and BA.2 neutralizing antibody titers among infected/vaccinated HCWs and trend curve (red line).

The median BA.1 neutralizing antibody (NAb) titer of the previously uninfected, vaccinated HCWs was 2 (IQR: 2–4) and their median anti-BA.2 NAb titer was 2 (IQR: 0–8) (Figure 2). The median anti-BA.1 titer in the infected-prior-to-vaccination HCWs was 8 (IQR: 4–16); it was 16 for BA.2 NAb (IQR: 16–32) (Figure 3). The correlation between the BA.1 and BA.2 neutralizing antibody titers was 0.78 (*p* < 0.01, Spearman test) among the previously uninfected, vaccinated HCWs and 0.86 (*p* < 0.01, Spearman test) among those infected before vaccination.

**Figure 4:**
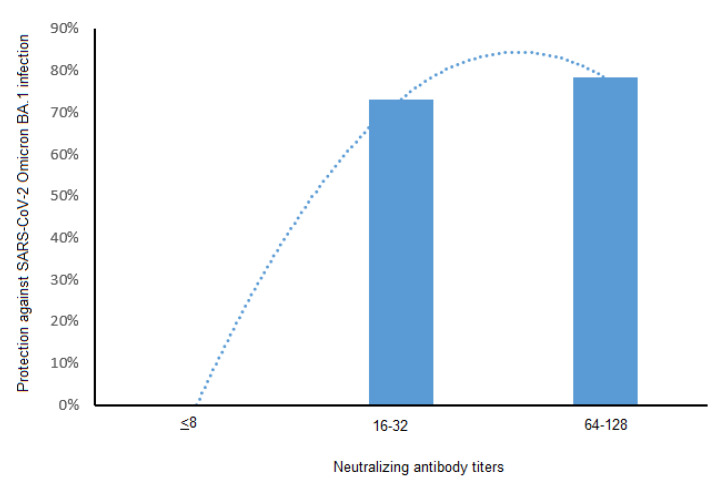
BA.1 neutralizing antibody titers and protection against SARS-CoV-2 Omicron BA.1 infection/re-infection with polynomial trend curve (dotted line).

About one third of the HCWs whose anti-BA.1 NAb titer was below 8 (average: 28.4%, 95% CI: 19.9–38.2%) became infected with Omicron BA.1. In contrast, few (7%, [95% CI: 1.5–19.1%]) of those whose BA.1 NAb titer was 16–32, or those with NAb titers of 64 or 128 (6.2%, [95% CI: 1.6–30.2%]) became BA.1 infected (*p* < 0.01, Chi^2^ test). An NAb titer below 8 provided no protection against a BA.1 infection, a titer of 16 or 32 gave 73.2% protection, and an NAb titer of 64 or 128 provided 78.4% protection (Figure 4). 

Conversely, the BA.2 infection rate did not vary as a function of anti-BA.2 NAb titers. Only 6.9% (95% CI: 2.8–13.6%) of the HCWs with an anti-BA.2 NAb titer below 8 became infected with Omicron BA.2, as did 16.3% [95% CI: 6.8–30.7%] of those whose BA.2 NAb titer was 16–32, and 22.2% [95% CI: 2.8–60%] of those with an anti-BA.2 NAb titer of 64 or 128 (*p* = 0.12, Chi^2^). 

### 3.4. Binding Antibodies and Protection against SARS-CoV-2 BA.1 and BA.2

**Figure 5:**
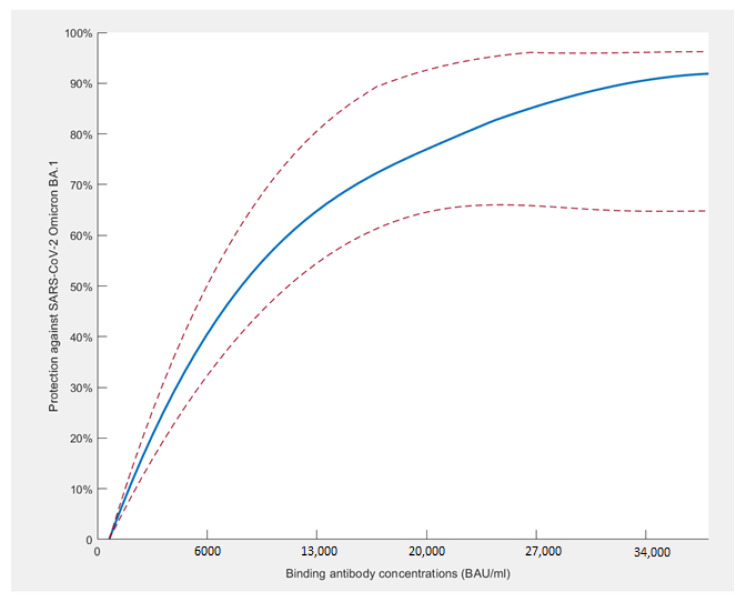
Binding antibody concentrations and protection against SARS-CoV-2 Omicron BA.1 infection/re-infection by spline interpolation (95% CI: dotted line).

The correlations between the binding antibody (BAb) concentrations and the BA.1 and BA.2 NAb titers were 0.71 (*p* < 0.01, Spearman test) and 0.89 (*p* < 0.01, Spearman test), respectively. Of those HCWs who became infected with BA.1 between November 2021 and March 2022, 26.9% [95% CI: 19.8–35.1%] had a BAb concentration below 6000 BAU/mL, 12.1% [95% CI: 3.4–28.2%] had a BAb concentration of 6000–20,000 BAU/mL, and only 3.3% [95% CI: 0.4–11.3%] had a BAb concentrations of 20,000 BAU/mL or more (*p* < 0.01, Chi^2^). BAb concentrations below 6000 BAU/mL provided no protection against Omicron BA.1 infection, 6000–20,000 BAU/mL provided 55.6% protection, and 20,000 or more provided 87.7% protection (Figure 5). 

On the other hand, there was no difference in BA.2 infection depending on the BAb concentration: 9.2% [95% CI: 5–15.2%] of HCWs with a BAb concentration below 6000 BAU/mL became infected with BA.2 between November 2021 and March 2022, as did 12.1% [95% CI: 3.4–28.2%] of those HCWs with a BAb concentration of 6000–20,000 BAU/mL, and 6.6% [95% CI: 1.8–15.9%] of those whose BAb concentration was 20,000 BAU/mL and above (*p* = 0.65, Chi^2^).

### 3.5. Antibody Concentrations and Vaccination Status

Half the previously-infected HCWs (10/20, 50%) given a booster dose had an anti-BA.1 NAb titer above 64, as did only 5/148 (3.4%) of those given two doses (*p* < 0.01, Fisher’s exact test). Those HCW given a booster dose (4/20, 20%) had BAb titers above 6000 BAU/mL, as did only 4/148 (2.7%) of those given two doses (*p* < 0.01, Fisher’s exact test). 

Only 1/75 (1.3%) of the HCWs who were not infected before vaccination and given a booster dose of vaccine had an anti-BA.1 NAb titer exceeding 64, or a BAb concentration above 6000 BAU/mL. None of the 16 vaccinated (2 doses, no booster) HCWs had a neutralizing antibody titer above 64 or a Bab concentration above 6000 BAU/mL.

## 4. Discussion

Several pre-oOmicron clinical [2,5,7,8,9] and animal [23] studies found that the neutralizing and binding antibody concentrations were good biomarkers of protection against a SARS-CoV-2 infection. We now find a similar relationship between the concentrations of anti-BA.1 binding and neutralizing antibodies and protection against a BA.1 SARS-CoV-2 infection. 

We also find that there is no clear antibody concentration above which Omicron infection does not occur, in contrast to infections with the alpha variant or other earlier strains (D614G) [7]. Although we found an inverse link between the risk of a SARS-CoV-2 BA.1 infection and the anti-BA.1 NAb and Bab concentration, there seems to be no such link for BA.2. This could be due to differences in the time between antibody assay and SARS-CoV-2 BA.1 and BA.2 infections, or differences in the restrictions in place during the BA.1 wave (November 2021–February 2022) and BA.2 wave (March 2022–May 2022). Wearing masks and physical distancing were abolished in France on 14 March 2022, which could have resulted in greater exposure to BA.2 infections, even in HCWs with high anti-BA.2 NAb and BAb concentrations. The degree of protection conferred on this same HCW population by a NAb against the B.1.160 strain (D614G) varied slightly [7]. Titers of 64–128 anti-B.1.160 NAb conferred 94% protection against a D614G strain, and 64–128 anti-BA.1 NAb conferred 78.4% protection against BA.1. While an anti-B.1.160 NAb titer of 256 or more provided 100% protection, no HCW had anti-BA.1 or anti-BA.2 NAb titers greater than 256. A BAb concentration of 141 BAU/mL or more provided approximately 89.3% protection against infection with a D614G strain, and a BAb concentration of 1700 BAU/mL or more provided 100% protection. Our present results indicate that a concentration of 20,000 BAU/mL is needed for an equivalent degree of protection, approximately 142-times the 141 BAU/mL threshold concentration. This is probably due to differences in the antigenicity of the spike proteins in Omicron BA.1 and the ancestral SARS-CoV-2 strain used in current vaccines and immunoassays [24]. Similarly, 8.5 times more binding antibody is required to neutralize the Delta variant than to neutralize the Alpha variant [14]. Our results partially agree with those of a recent Danish study [25] that demonstated that the inverse relationship between the IgG level and the risk of contagion observed for the Delta variant demonstrated the protective effect of vaccines, but this association was not observed for the Omicron variant. This suggests that the quantitative level of anti-spike IgG has a limited impact on the risk of breakthrough infection with Omicron. Note, however, that the study did not distinguish between infections caused by the BA.1 strain or the BA.2 strain of Omicron. Another study on nursing home residents demonstrated correlates of protection between the level of (binding and neutralizing) antibodies and Omicron BA.2 infection [26]. A BA.2 NAb title below 8 provided 19.2% protection against Omicron BA.2, titles of 16 or 32 gave 85.3% protection and an NAb title of 64 or more provided 95.6% protection. A BAb concentration below 1000 BAU/mL provided only 25% protection against Omicron BA.2; 1000–6000 BAU/mL provided 77.9% protection; while >6000 provided 97.1% protection. It should be noted, however, that this population, which is particularly at risk, continued to benefit from restrictive sanitary measures, unlike the general population, and that in this study it was very little exposed to Omicron BA.1.

Although current vaccines reduce the risk of a symptomatic SARS-CoV-2 infection, hospitalization and death, their effectiveness against the Omicron variant [11,17,27,28] is altered due to lower neutralizing antibody titers. We found that a greater percentage of previously infected, vaccinated HCWs had high NAb and BAb titers than did uninfected vaccinated HCW. This is consistent with studies indicating that vaccination of previously infected individuals gives greater protection than the vaccination of naive individuals [29]. 

The limitations of this study concern both vaccination and antibody measurement. First, antibodies were not measured at the time of SARS-CoV-2 breakthrough. Waning antibody titers are associated with loss of protection [30] and the post-vaccination total antibody titer decreases faster in vaccinated people with no previous SARS-CoV-2 infection than in previously-infected, vaccinated individuals [30]. Second, all the HCWs were not given a booster vaccination at the time of analysis, particularly those HCWs who were infected before being vaccinated. The majority were given a complete (2-dose) schedule without booster. Third, we did not discriminate between infection and symptomatic disease. The antibody concentrations providing robust protection from severe infection require further investigation in other populations. Last, the contribution of the T-cell system to protection was not assessed.

## 5. Conclusions

This is, to our knowledge, the first study of protective antibody thresholds against infection with Omicron BA.1 and BA.2. We provide further evidence in a longitudinal cohort that robust antibody levels against the ancestral strain fail to establish sufficient protection against antigenically distant variants. Further, we demonstrate that a full booster vaccination schedule without infection may not adequately confer protection against breakthrough infection with the Omicron variant [25]. In order to achieve more robust immune protection and limit SARS-CoV-2 transmission, the next generation of SARS-CoV-2 vaccine preparations will include Omicron subvariant’s spike determinants in addition to ancestral strain antigens [31].

While our results provide markers of protection against BA.1 infection, further studies are needed to investigate the relationship between antibody concentrations and infection with the Omicron BA.4/5 variants that are becoming predominant worldwide. 

## Figures and Tables

**Table 1:** HCW characteristics according to infectious status: infected then vaccinated or vaccinated without a past infection.

HCWs	Infected/Vaccinated	Vaccinated
N (%)	168 (64.9)	91 (35.1)
Age:		
Median (IQR)	39 (21–58)	42 (21–61)
Female: N (%)	130 (77.4)	63 (69.2)
Vaccines: *n* (%)		
BNT162b/BNT162b	152 (90.5)	37 (40.7)
ChadOx1-S/BNT162b	16 (9.5)	46 (50.5)
ChadOx1-S/ChadOx1-S	0	8 (8.8)
Vaccination status: *n* (%)		
-Primary vaccinated (2 doses)	148 (88.1)	16 (17.6)
-Primary vaccinated + booster dose	20 (11.9)	75 (82.4)

## Data Availability

The data that support the findings of this study are available from the corresponding author upon reasonable request.
